# Energy Metabolism in CKD: Running Low on Fuel

**DOI:** 10.34067/KID.0000000000000231

**Published:** 2023-08-31

**Authors:** Marie Christelle Saade, Samir M. Parikh

**Affiliations:** Division of Nephrology, Department of Medicine, University of Texas Southwestern, Dallas, Texas

**Keywords:** CKD, chronic kidney failure, metabolism

CKD is characterized by the gradual loss of glomerular filtration function over time. Yet, emerging evidence suggests that CKD is accompanied by intrarenal impairments that extend far beyond filtration. One area that has received substantial attention is the profound alteration in intrarenal metabolism during CKD. These metabolic disturbances may contribute to disease progression.^1^ Understanding and addressing these metabolic alterations is critical for unraveling the complex pathophysiology of CKD and developing targeted interventions to alleviate its burden. In this issue of *Kidney360*, Li and colleagues report a combination of transcriptomics, metabolomics, and targeted assays interrogating metabolism in a subtotal nephrectomy (STN) murine model of CKD.^2^

The authors focused on a time point 4 weeks after STN when GFR had declined by more than 60% of sham controls (approximately 400–150 *μ*l/min). Bulk whole-transcriptome analysis revealed 872 genes displaying significant differential expression, with 470 genes being overexpressed and 402 genes being underexpressed in STN kidneys compared with sham kidneys. Significant alterations were observed in the regulation of mitochondrial function, fatty acid metabolism, and glucose metabolism.

The most significantly different biological pathway was oxidative phosphorylation, within which most of the genes were upregulated in STN kidneys. Furthermore, transcripts for two major substrate utilization pathways used for energy generation—fatty acid oxidation (FAO) and glycolysis—were upregulated in the STN kidneys compared with sham kidneys. In fact, the mRNA for the rate-limiting enzymes in FAO, carnitine palmitoyltransferase I and II, as well as the mRNA levels of several glycolytic enzymes, including hexokinases 1 and 2, phosphofructokinase, and pyruvate kinase, were significantly increased in STN kidneys compared with sham kidneys. The mitochondrial copy number, reflecting mitochondrial content, and the expression of peroxisome proliferator-activated receptor gamma coactivator1 alpha (Pgc1*α*), reflecting mitochondrial biogenesis, were both increased in STN kidneys. The increase in Pgc1*α* is notable because this gene's induction has been shown in several studies to promote the health of the renal tubule in the setting of acute stress or inducers of fibrosis.^3–5^ These results suggest a model in which the remnant tubule mass increases its mitochondrial mass to respond to the higher solute load arising from increased single-nephron GFR.^6^

On the other hand, Li and colleagues found that the end product of glucose catabolism, pyruvate, was reduced in STN kidneys. Pyruvate is normally either converted to acetyl CoA, for entry into the Krebs cycle, or to lactate, which permits ongoing flux through glycolysis and the recycling of nicotinamide adenine dinucleotide hydrogen back to nicotinamide adenine dinucleotide. However, both acetyl CoA and lactate were reduced in STN kidneys. Notably, the activity of pyruvate dehydrogenase (PDH), which catalyzes the conversion of pyruvate to acetyl CoA, was revealed to be intact, but the protein expression of the PDH E1 subunit and the activity of PDH phosphatase, which activates PDH, were both identified as reduced in STN kidneys.

Making the metabolic consequences of nephron mass reduction even more complex to discern, Li and colleagues found that functional assays in freshly isolated proximal tubules displayed a consistent profile of mitochondrial impairment. Specifically, mitochondrial basal and maximum oxygen consumption rates and respiratory control ratio, coupling efficiency and ATP-linked respiration to maximal respiration, were all decreased in proximal tubules isolated from STN kidneys compared with sham control counterparts. Conversely, measures of glycolysis in this same experimental setup revealed either increased or unchanged flux in proximal tubules isolated from STN kidneys compared with controls. Together, the flux assays implicated decreased mitochondrial function—despite the elevated oxidative phosphorylation signature in whole-kidney RNA sequencing, increased mitochondrial DNA copy number, and induction of Pgc1*α*—in STN kidneys' proximal tubules with a concomitant increase in glycolytic flux.

As the authors note, some of this discrepancy could relate to substrate utilization: For example, the per-cell mitochondrial mass may indeed be elevated in the proximal tubule after STN, but defects in substrate import or key steps in substrate conversion to fuel might impair actual oxidative phosphorylation in mitochondria. Although technical issues could impair the fidelity of functional assays, freshly isolated tubules should maintain the core metabolic functions they possessed in vivo. Another possibility is that the whole-kidney RNA sequencing and metabolomics reflect compensatory changes against contrary events in the proximal tubule, *e.g.*, a shift to aerobic glycolysis in the proximal tubule swamped out by a shift to mitochondrial metabolism in many other compartments of the STN kidney. Finally, as the authors caution, metabolomics offers a static snapshot of intermediary metabolism reflecting the balance of anabolic and catabolic processes, whereas flux studies help address the actual flow of substrates.

**Figure 1:**
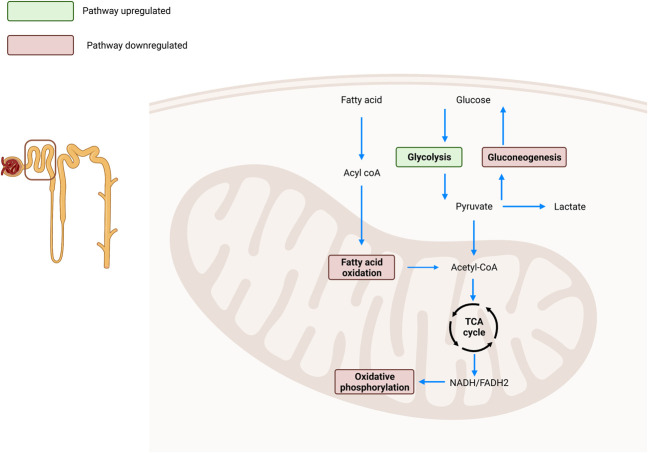
**The regulation of metabolic pathways within proximal tubular cells during the CKD.** The glycolysis pathway is described as upregulated in CKD (shown in green). Gluconeogenesis, fatty acid oxidation, and oxidative phosphorylation are described as downregulated in CKD (shown in red). NADH, nicotinamide adenine dinucleotide hydrogen; FADH2, (di)hydroflavin flavin dinucleotide

Several previous studies have emphasized the importance of understanding metabolic adaptations in CKD.^7^ Nonetheless, Li and colleagues have provided, to our knowledge, the first extensive description of metabolic alterations in the mouse STN setting, a model that addresses the reduced nephron mass of CKD.^8^ Glycolysis, FAO, and gluconeogenic pathways were found to be transcriptionally upregulated by Li *et al.* 4 weeks after STN surgery. The upregulation of glycolysis in CKD is consistent with the existing literature, in contrast to FAO and gluconeogenesis, which are typically described as downregulated^9^ (Figure 1). In proximal tubular cells, CKD progression is usually associated with a switch from FAO to glycolysis in a stage-dependent manner. Depressed renal tubular FAO has even been proposed to be pathogenic in CKD on the basis of genetic models restoring fatty acid import into mitochondria that result in decreased fibrosis after experimental injury.^5^ Conversely, inhibition of glucose import into the proximal tubule by sodium glucose linked cotransporter inhibitors confers extraordinary renoprotection against a wide range of CKD etiologies.^10^

These results highlight the complexity of metabolic regulation in progressive kidney disease and the need for the type of complementary approaches undertaken by Li and colleagues. Further spatial resolution of transcriptomic and metabolic changes, extended through multiple time points in the transition from acute injury to chronic fibrosis and perhaps even across different experimental models, may illuminate the complex spatiotemporal regulation of metabolism and novel opportunities to develop noninvasive health and disease markers or even metabolically inspired treatments. A recent study by Wang and colleagues highlights the promise of coregistered spatial transcriptomics and metabolomics to open such new avenues in kidney biology.^11^

To conclude, Li and colleagues report an extensive metabolic characterization of a model of reduced nephron mass. Their results highlight a shift away from mitochondrial oxidative metabolism and toward glycolysis in the proximal tubule, despite the increased solute load encountered by remnant nephrons in this model. Further research should further unravel the dynamics and regional heterogeneity of metabolic alterations in CKD. Given the unexpected and outsized success of sodium glucose linked cotransporter inhibitors against CKD progression, there is an urgent need to advance our knowledge of metabolic processes in kidney health and disease.
